# Engineering pedicled vascularized bladder tissue for functional bladder defect repair

**DOI:** 10.1002/btm2.10440

**Published:** 2022-10-29

**Authors:** Mingming Yu, Jiasheng Chen, Lin Wang, Yichen Huang, Hua Xie, Yu Bian, Fang Chen

**Affiliations:** ^1^ Department of Urology Shanghai Children's Hospital, Shanghai Jiao Tong University Shanghai China; ^2^ Department of Urology Shanghai Jiao Tong University Affiliated Sixth People's Hospital Shanghai China; ^3^ Shanghai Eastern Urological Reconstruction and Repair Institute Shanghai China; ^4^ Department of Ultrasound in Medicine Shanghai Jiao Tong University Affiliated Sixth People's Hospital Shanghai China

**Keywords:** bladder, buccal mucosa, capsule, cell sheets, tissue engineering, vascularization

## Abstract

An engineered bladder construct that mimics the structural and functional characteristics of native bladder is a promising therapeutic option for bladder substitution. We previously showed that pedicled vascularized smooth muscle tissue fabricated by grafting smooth muscle cell (SMC) sheets onto an axial capsule vascular bed had the potential for reliable bladder reconstruction. In this study, we investigated the feasibility of buccal mucosa graft (BMG) integration with the pedicled vascularized smooth muscle tissue to generate a full‐layer pedicled vascularized bladder construct. BMG transplanted onto vascularized smooth muscle tissue showed good survival and developed into a pedicled vascularized bladder construct with full‐layer structures, appropriate thickness, abundant vascularization, and effective barrier function. Then the full‐thickness bladder defects were, respectively, reconstructed by pedicled capsule tissue (pedicled capsule group), nonpedicled vascularized bladder construct (nonpedicled construct group), and pedicled vascularized bladder construct (pedicled construct group). The picrosirius red (PSR) staining and immunohistochemistry results showed minimal fibrosis, maximal smooth muscle proportion, and high vascular density in the pedicled construct group. A continuous mucosal layer was observed only in the pedicled construct group. Moreover, morphological and functional studies revealed better bladder compliance and good ductility in the pedicled construct group. Overall, these results suggested that the BMG could be well integrated with vascularized smooth muscle tissue and generated a pedicled, fully vascularized, and structurally organized bladder construct, which facilitated structural remodeling and functional recovery and could become an alternative to bladder reconstruction.

## 1 INTRODUCTION

Tissue engineering has been proven to be a promising alternative for gastrointestinal segments in clinical bladder reconstruction over the past few decades.^1^ Although great achievements have been made in preclinical researches, the clinical applications encounter a series of problems, leading to unsatisfactory results.^2^, ^3^, ^4^, ^5^, ^6^ This may be mainly attributed to underestimation of the bladder, and many challenges remain to be solved.^7^, ^8^

Vascularization remains a major challenge in tissue engineering, especially for large and thick tissues such as bladder tissue.^9^ Sufficient and timely blood supply is the basis of survival and growth, which has not been effectively achieved in clinical practice.^10^ Thus, the results of clinical applications are usually related to severe fibrosis and poor function. Developing a vasculature to solve the problem of tissue oxygenation and nutrient/waste exchange is increasingly being recognized as a major issue.^11^ However, traditional approaches, such as the application of angiogenic agents and vascular endothelial cells, can hardly work in large or thick grafts.^12^, ^13^ This is mainly because the implanted graft still needs to establish a vascular connection with the host at a low rate of approximately 5 μm/h.^14^ In recent years, fabricating vascularized constructs with vascular pedicles has been proven to be an effective strategy.^15^, ^16^ In our previous studies, we induced capsule tissue with superficial circumflex iliac vessels (SCIs) as its axial vessels and demonstrated the feasibility of making it a novel vascular bed in vivo to generate pedicled vascularized tissues for urinary reconstruction.^17^, ^18^, ^19^ Recently, we used the capsule vascular bed to cultivate smooth muscle cell (SMC) sheets and fabricate pedicled vascularized smooth muscle constructs for reliable bladder detrusor reconstruction.^20^ On this foundation, we intended to generate multilayered constructs for full‐thickness bladder reconstruction.

Bladder tissue has a complex structure, and the urothelium and detrusor are the most fundamental components.^21^ The mucosal layer of the engineered construct should have an effective barrier function before implantation, as hypertonic and cytotoxic urine is believed to exert an adverse impact on inner cells.^22^ However, this has not been achieved in most relevant studies.^23^ Cell‐based strategies seem to be unsuitable for clinical application. Expansion of epithelial cells in vitro is somewhat difficult and usually requires feeder cells such as 3 T3 fibroblasts, which may inevitably lead to mixing with allogeneic or heterogeneous cells. Buccal mucosa, which possesses barrier function and is widely used for clinical urethral reconstruction, has been proven to be a suitable material for bladder mucosa reconstruction.^24^ Thus, from the point of clinical application, we selected buccal mucosa as the source of the mucosal layer and investigated the feasibility of integrating BMG with the engineered vascularized smooth muscle tissue to generate a full‐layer bladder construct.

**FIGURE 1:**
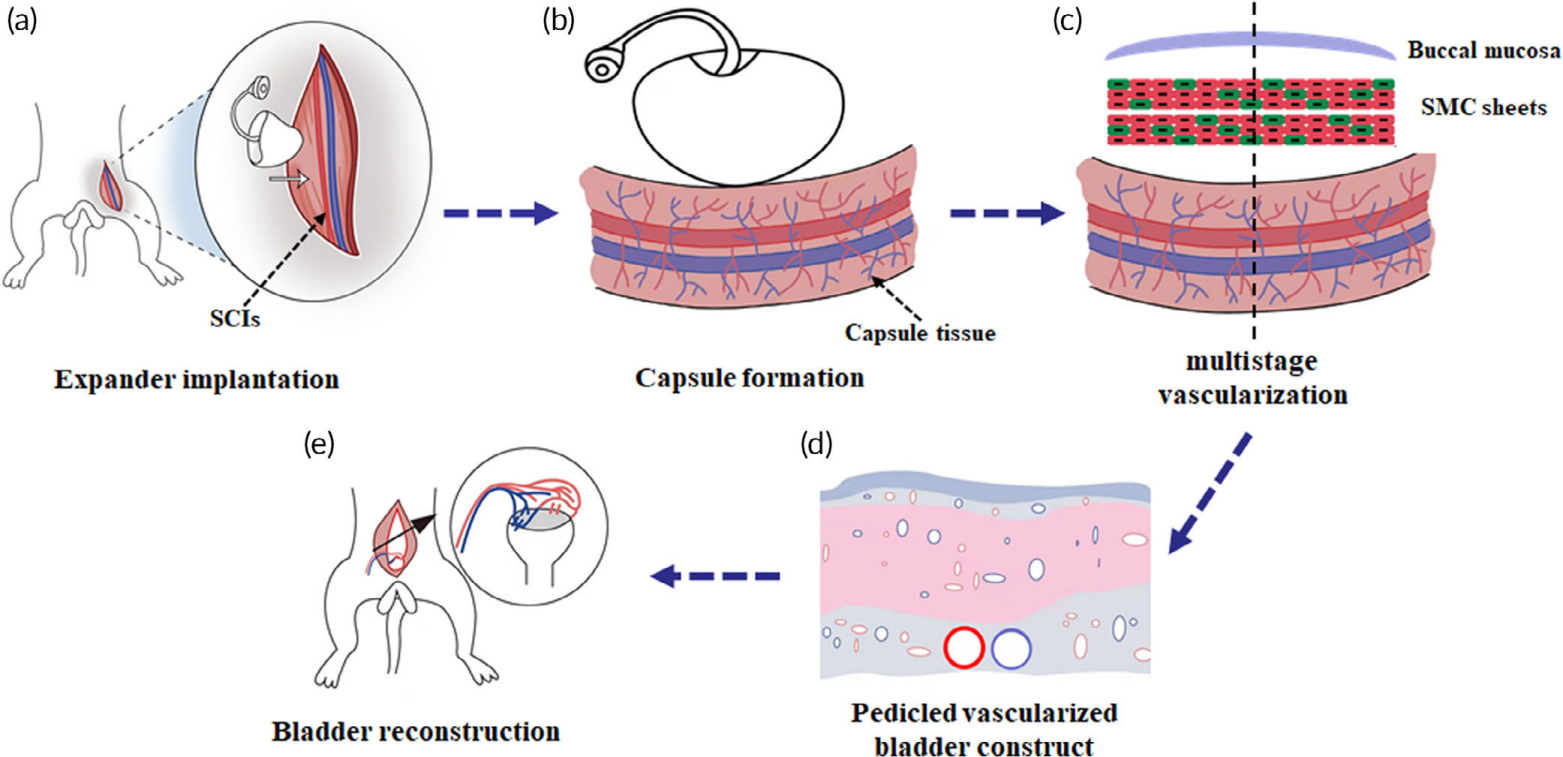
Experimental workflow. (a) A skin expander was implanted adhere to the separated superficial circumflex iliac vessels (SCIs) in the inguinal region and then injected with normal saline at 2‐day intervals until fully filled. (b) The capsule tissue was induced after full expansion of the expander. (c) Six‐layer smooth muscle cell sheets were transplanted on the capsule vascular bed in twice at a 2‐day interval for vascularization. Then a buccal mucosa graft (BMG) was transplanted on the vascularized smooth muscle tissue to fabricate composite bladder tissue. (d) The BMG integrated well with the vascularized smooth muscle tissue and developed into full‐layer vascularized bladder tissue with SCIs as its vascular pedicle. (e) The vascularized bladder tissue was transferred from inguinal region to abdominal cavity along with its vascular pedicle for bladder reconstruction.

Here, we reported for the first‐time development of a full‐layer pedicled vascularized bladder construct engineered by combining BMG with an engineered vascularized smooth muscle construct. A pedicled vascularized smooth muscle construct was generated by grafting six‐layer SMC sheets onto the axial capsule tissue in two steps. BMG with partial submucosa transplanted onto the smooth muscle construct showed good integration and maintenance of barrier function. A rabbit full‐thickness bladder defect model made by partial cystectomy, a well‐used model for bladder reconstruction, was used in this study. Then the pedicled vascularized bladder construct was used for bladder reconstruction and the results revealed a consecutive and ductile mucosal layer, an appropriate smooth muscle proportion with decreased fibrosis and good compliance. Overall, our approach provided a fully vascularized and structurally organized bladder substitute, which could become a novel and effective alternative for clinical bladder reconstruction (Figure 1).

## 2 RESULTS

### 2.1 Characteristics of capsule tissue

**FIGURE 2:**
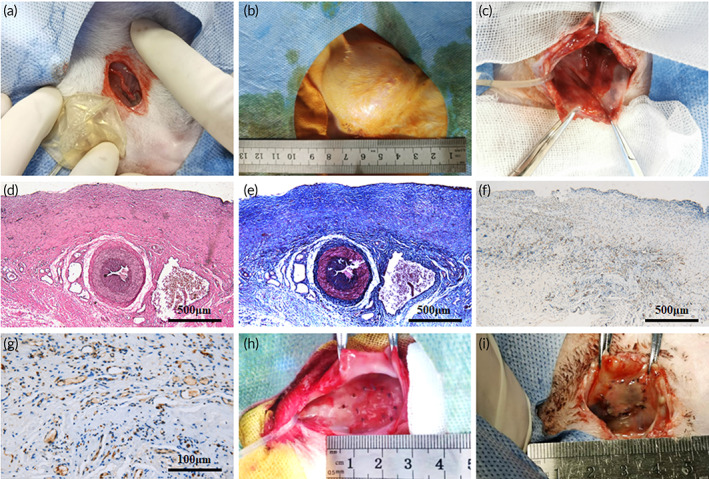
Induction of the capsule tissue and multistage bioengineering of pedicled vascularized bladder construct. (a) A skin expander (15 ml) was implanted close to the separated superficial circumflex iliac vessels (SCIs) in the inguinal region. (b) The skin expander was fully injected with normal saline. (c) Gross appearance of the capsule tissue. (d–e) HE staining and Masson staining of the capsule tissue showed that the fibrous capsule tissue consisted of three layers: cellular layer (closest to the expander), central layer, and fibrous layer. (f) Immunohistochemical staining for CD31 showed numerous microvessels in the capsule tissue. (g) A magnification of image (e). (h) The six‐layer SMC sheets grew into vascularized smooth muscle tissue after 7 days of cultivation on the capsule vascular bed. (i) The buccal mucosa graft integrated well with the vascularized smooth muscle tissue and formed into a composite vascularized bladder construct after transplanting and cultivating for another 7 days.

The capsule tissue was induced by a round skin expander (spherical, 15 ml) adhered to the separated SCIs in the inguinal region (Figure 2a) and then intermittently injected with normal saline until fully expanded (Figure 2b). Macroscopically, the capsule tissue had a hollow structure and smooth surface. Inside the capsule tissue were the pulsatile SCIs, which sprouted abundant microvessels extending to the periphery (Figure 2c). The results of HE staining and Masson staining demonstrated that the capsule tissue was comprised of cellular layer (closest to the expander), central layer (containing the SCIs), and fibrous layer (Figure 2d–e). Immunohistochemical staining for CD31 showed numerous microvessels within the capsule tissue (Figure 2f,g).

### 2.2 Fabrication of vascularized bladder construct

The six‐layer SMC sheets were successfully transplanted onto the in vivo vascular bed in two steps and grew into vascularized smooth muscle tissue (Figure 2h). Then, the BMG was transplanted onto the vascularized smooth muscle tissue and cocultivated for another 7 days. Finally, the BMG and six‐layer SMC sheets integrated into a pedicled vascularized bladder construct with an average diameter of 1.8 cm (Figure 2i).

### 2.3 Angiogenesis of smooth muscle tissue

**FIGURE 3:**
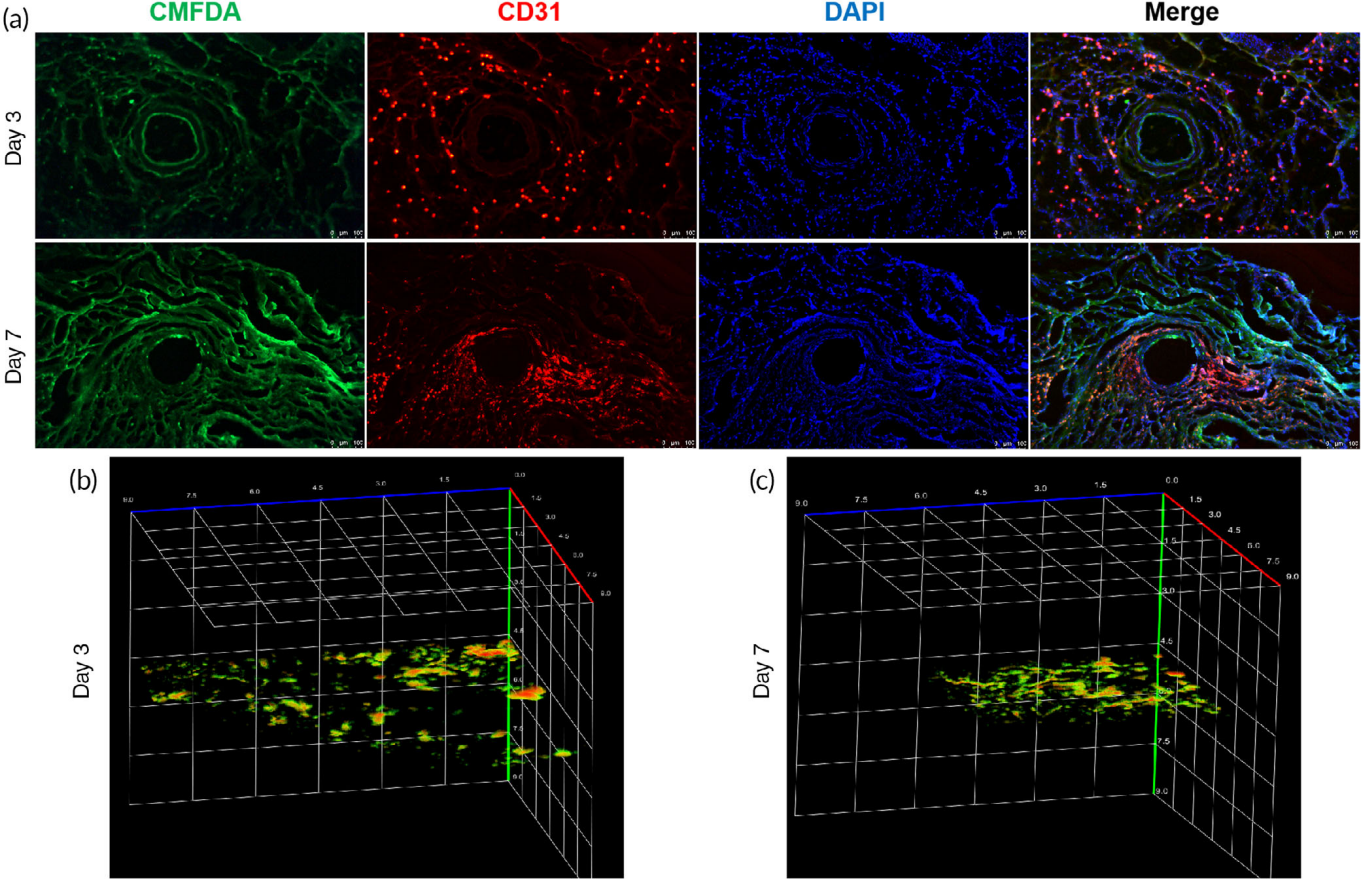
Angiogenesis assessments of the vascularized smooth muscle tissue and the contribution of EPCs to angiogenesis. (a) The immunofluorescence staining for CD31 of the vascularized smooth muscle tissue indicated that the EPCs prelabeled with CellTracker Green CMFDA formed a confluent network structure and CD31 negative tube‐like structure after 3 days of cultivation, and a more intensive network structure and CD31 positive microvessels consisting of partial prelabeled EPCs were observed after 7 days of cultivation. (b, c) PAM scanning showed that only a few separative vascular vessels sprouted in the smooth muscle tissue after 3 days of cultivation and more vessels with mature structures were seen after 7 days. EPCs, endothelial progenitor cells; SMCs, smooth muscle cells; PAM, photoacoustic microscope

The EPCs used for fabricating cell sheets were prelabeled with CellTracker Green 5‐Chloromethylfluorescein Diacetate (CMFDA). The immunofluorescence of the cultivated smooth muscle tissue indicated that the EPCs formed tube‐like structures and confluent network structures after 3 days of cultivation on a capsule vascular bed (Figure 3a). After 7 days of cultivation, the network structures were enhanced, and the EPCs participated in the formation of CD31‐positive microvessels (Figure 3a). The photoacoustic microscope (PAM) results showed that only a few separative vascular vessels sprouted in the smooth muscle tissue after 3 days of cultivation (Figure 3b), and more vessels with mature structures were seen after 7 days (Figure 3c).

### 2.4 Histological characteristics of the vascularized bladder construct

**FIGURE 4:**
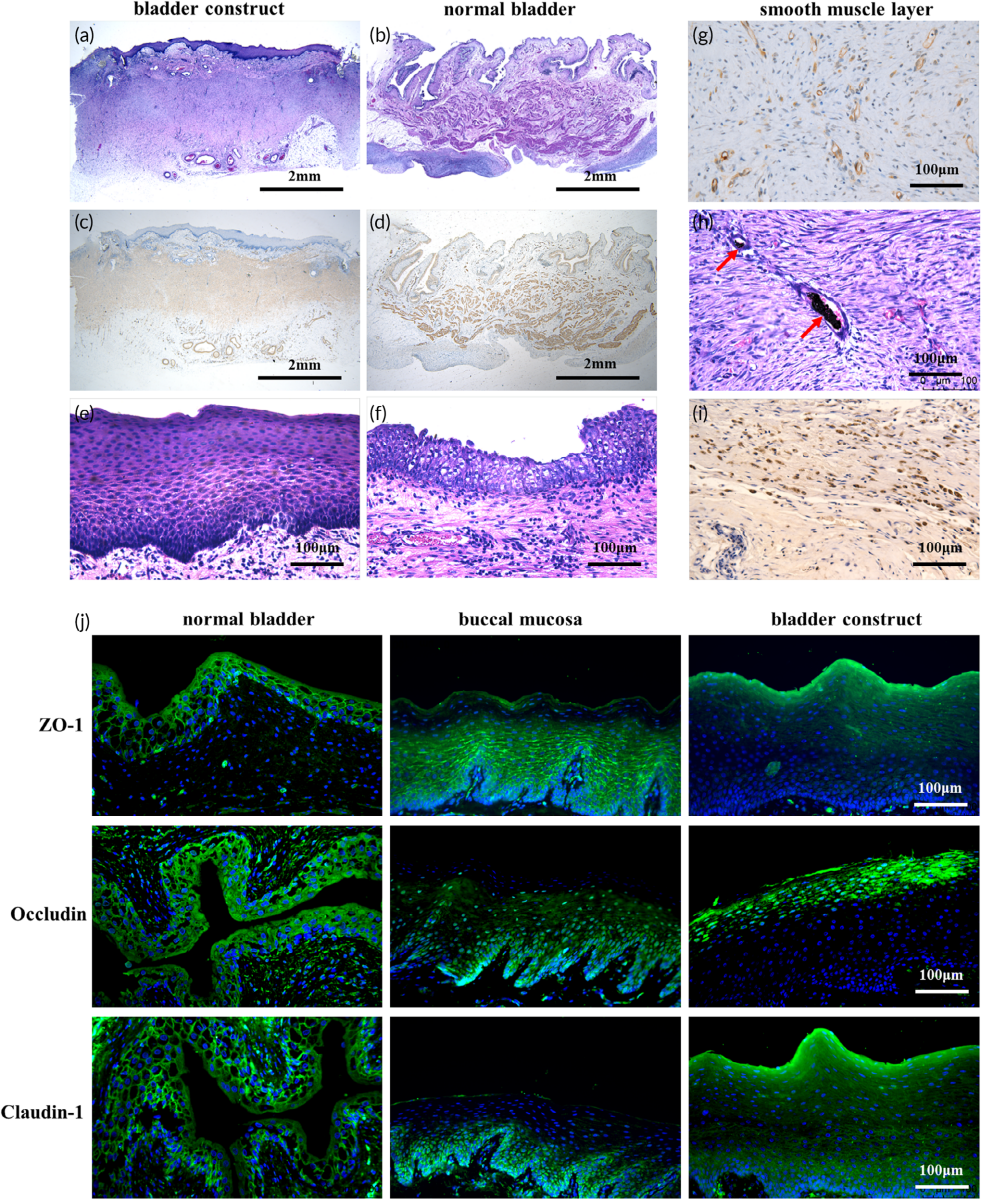
Histological characteristics of the vascularized bladder construct. (a, b) HE staining of the vascularized bladder construct and normal bladder tissue. (c, d) Immunohistochemical staining for α‐SMA of the vascularized bladder construct and normal bladder tissue. (e, f) Mucosa layer of the vascularized bladder construct and normal bladder tissue. (g) Immunohistochemical staining for CD31 showed numerous microvessels in the smooth muscle layer of the vascularized bladder construct. (h) Indian ink injection study demonstrated that the many blood vessels within the vascularized construct were filled with black ink. (i) Immunohistochemical staining for Ki67 revealed many Ki67 positive cells in the smooth muscle layer of the vascularized bladder construct. (j) Immunofluorescence staining for the key barrier function markers ZO‐1, occludin, and claudin‐1 in normal bladder tissue, buccal mucosa and bladder construct. Red arrows: India ink perfused vessels

According to the HE staining, the bladder construct with an average thickness of 3 mm (Figure 4a), similar to normal bladder tissue (Figure 4b), could be generally divided into four layers: mucosal layer, submucosal layer, smooth muscle layer, and capsule tissue layer. The smooth muscle layer was consecutive and uniform (Figure 4c). Its average thickness was approximately 1.5 mm, which was slightly thinner than that in normal bladder tissue (Figure 4d). The mucosal layer was even and consecutive, with an average thickness of 0.2 mm (Figure 4e), which was twice as thick as the normal bladder mucosa (Figure 4f). In addition, numerous microvessels in the smooth muscle layer were observed in the smooth muscle layer of the bladder construct according to immunohistochemical staining for CD31 (Figure 4g) and the Indian ink injection study showed that the many blood vessels within the vascularized construct were filled with black ink, indicating that the blood supply of the construct was derived from the axial artery (Figure 4h). Furthermore, a large portion of cells in the smooth muscle layer of the bladder construct were Ki67 positive, indicating a good proliferative state (Figure 4i). At last, the mucosal layer of the construct maintained its barrier function with continuous expression ZO‐1, claudin‐1, and occludin, similar to native bladder mucosa (Figure 4j).

### 2.5 Bladder reconstruction

**FIGURE 5:**
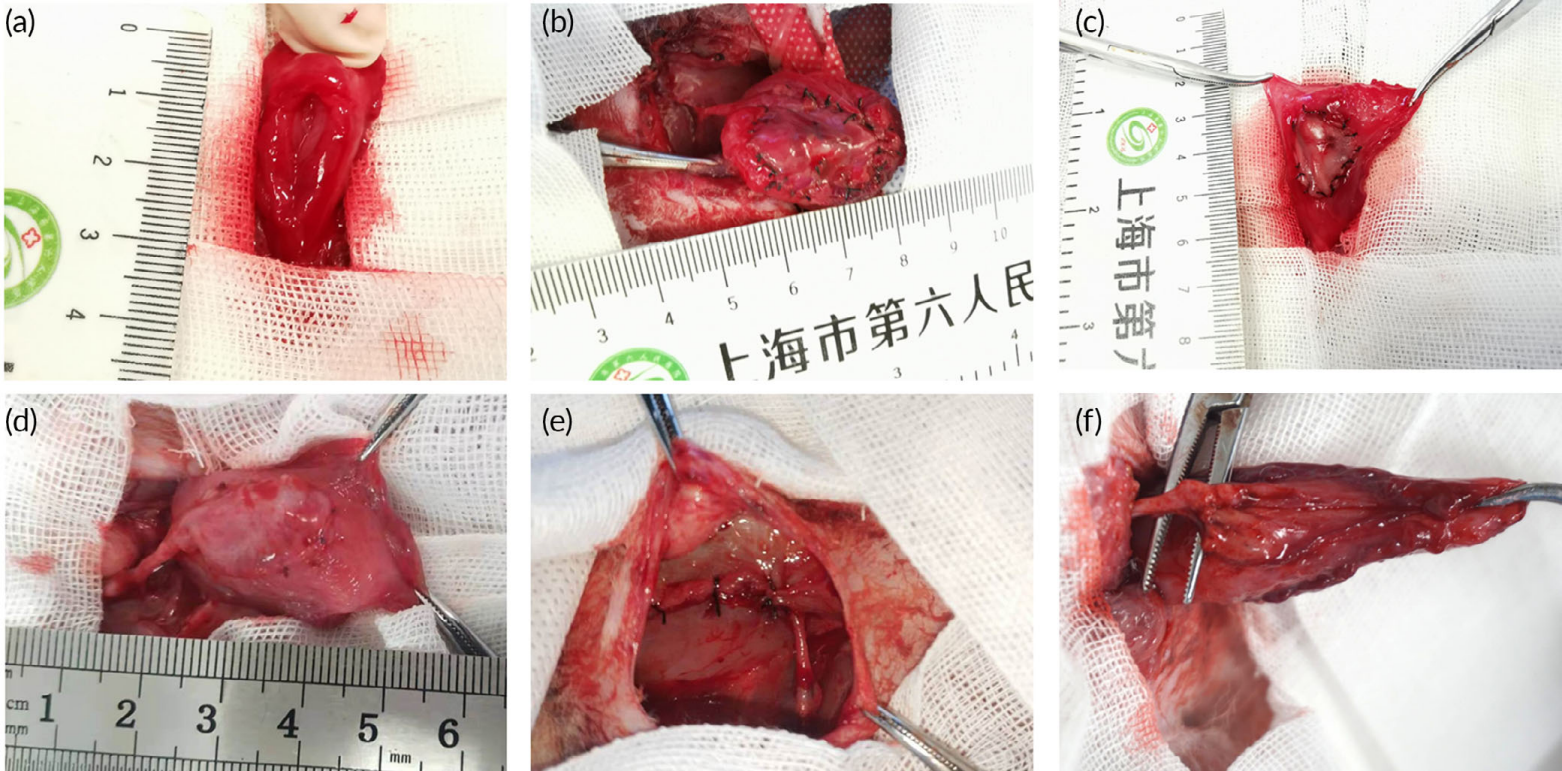
Bladder reconstruction. (a) A bladder defect withwith a mean diameter of 1.8 cm was created by a full‐thickness resection on the anterior wall of the bladder in an empty state. (b–d) The defective bladders were reconstructed, respectively, by pedicled capsule tissue flap, free vascularized bladder tissue flap and pedicled vascularized bladder tissue flap with corresponding size. (e) The pedicled flaps entered the abdominal cavity through an incision on the ipsilateral abdominal wall. (f) The vascular pedicle remained complete and connective during the follow‐up of 3 months.

A full‐thickness bladder defect was successfully created on the anterior bladder wall in an empty state (Figure 5a). The area of the bladder defect was approximately 2.5 cm^2^ and accounted for 25%–30% of the total area of a whole rabbit bladder (exclusive of the bladder neck), which was approximately 9–10 cm^2^ according to our measurement. Then, the defective bladders were, respectively, repaired by pedicled capsule tissue, nonpedicled vascularized bladder construct, and pedicled vascularized bladder construct (Figure 5b–d). The pedicled flaps entered the abdominal cavities through an incision on the ipsilateral abdominal wall (Figure 5e). During the follow‐up of 3 months, the vascular pedicle remained complete and connective (Figure 5f). In addition, all the rabbits were able to urinate spontaneously. No signs of infection, urine leakage, or bladder rupture were found during the follow‐up.

### 2.6 Histological characteristics of the reconstructed bladders

**FIGURE 6:**
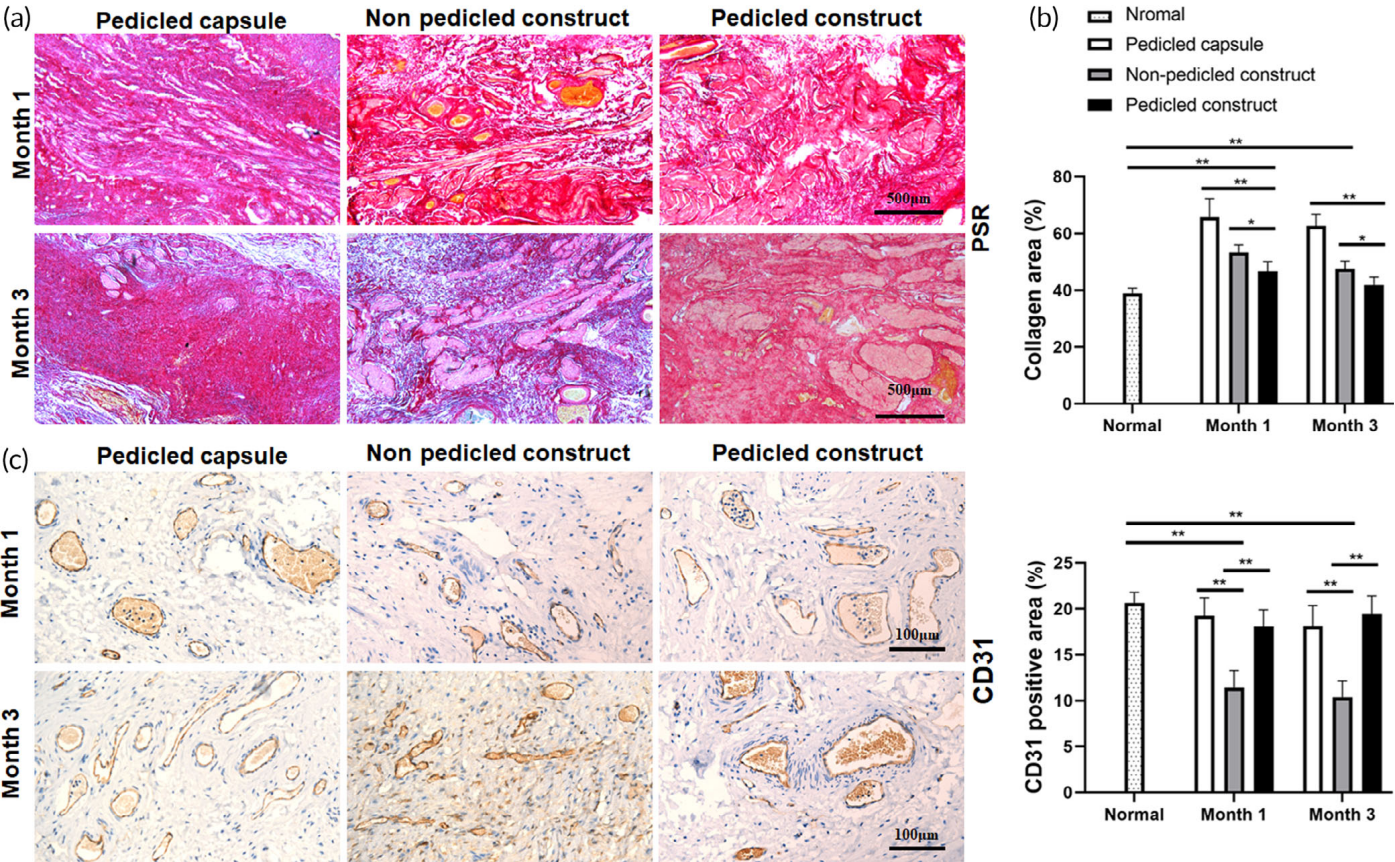
Fibrosis and vascularization of the reconstructed bladders. (a) PSR staining of the reconstructed bladders at 1 and 3 months after reconstruction. (b) Quantification of the collagen content. (c) Immunohistochemical staining for CD31 of the reconstructed bladders at 1 and 3 months after reconstruction. (d) Quantification of CD31 positive area. The data are shown as the mean ± SD; **p < 0.01, *p < 0.05.

The histological characteristics of the reconstructed bladders were assessed by PSR staining and immunohistochemical staining. The PSR staining revealed the maximal proportion of collagen in the pedicled capsule group and the minimum in the pedicled construct group (Figure 6a,b). Although a decline trend was observed in all groups, the collagen contents were all significantly higher than normal bladder tissue except for that in the pedicled construct group at 3 months. The CD31‐positive microvessels were significantly higher in the pedicled capsule group and pedicled construct group, which were close to normal bladder tissue, than in the nonpedicled construct group at both 1 and 3 months (Figure 6c,d). This might be attributed to the presence of a vascular pedicle.

**FIGURE 7:**
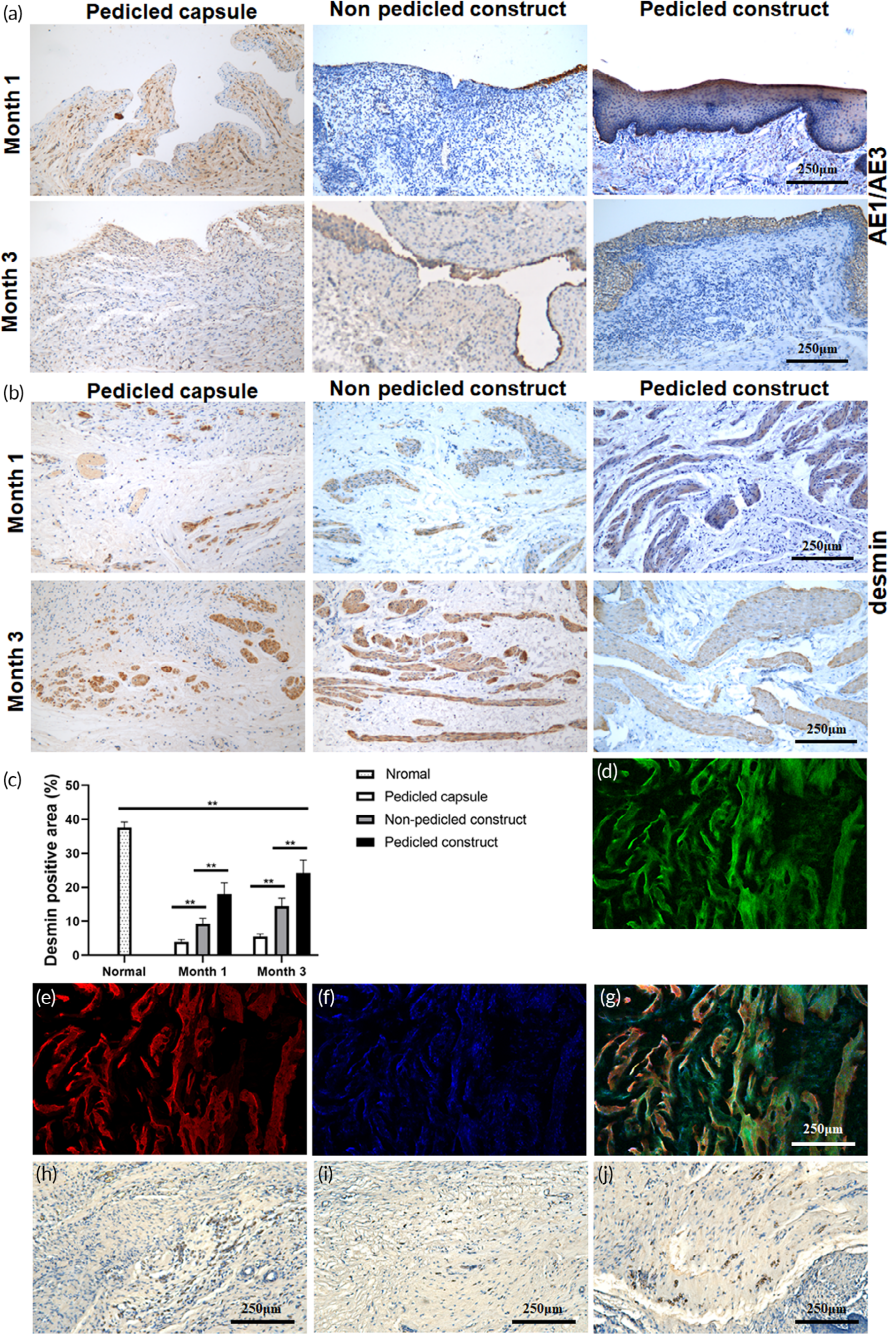
Histological examination of the mucosa layer and smooth muscle layer of the reconstructed bladders. (a) Immunohistochemical staining for AE1/AE3 of the reconstructed bladders at 1 and 3 months after reconstruction. (b) Immunohistochemical staining for desmin of the reconstructed bladders at 1 and 3 months after reconstruction. (c) Quantification of the smooth muscle proportion. (d–g) Immunofluorescence for desmin of the reconstructed bladders in the pedicled construct group. (d) CMFDA‐labeled cells (green), (e) Desmin positive SMCs (red), (f) Nuclei labeled with DAPI (blue), (g) merged image. (h–j) Immunohistochemical staining for S100β in the pedicled construct group. Positive nerve cells in the smooth muscle layer (h), capsule tissue layer (i), and submucosa layer (j). The data are shown as the mean ± SD; **p < 0.01.

There was only sporadic epithelial tissue and smooth muscle tissue in the pedicled capsule group after reconstruction (Figure 7a,b), which were considered as the results of cell migration and proliferation from adjacent normal bladder tissue. In the nonpedicled construct group and pedicled construct group, the implanted sites were approximately 2.5–3 mm in thickness, while the mucosal layers were consecutive in the pedicled construct group but poor in the nonpedicled construct group (Figure 7a).

The smooth muscle content in the pedicled capsule group and nonpedicled construct group was significantly lower than that in the pedicled construct group at both 1 and 3 months (Figure 7b,c). Well‐organized smooth muscle tissues were observed in the pedicled construct group at 3 months (Figure 7b). However, the smooth muscle content and cell density in the smooth muscle layer were all lower than the normal bladder tissue, especially in the pedicled capsule group and nonpedicled construct group. The proportion of smooth muscle only ranged from about 4% to 25% in each group, while it was appropriately 40% in normal bladder tissue. Fortunately, an increasing trend in smooth muscle content was apparently seen in both nonpedicled construct group and pedicled capsule group, which might be attributed to good proliferation of the implanted SMCs. To prove the effects of the SMC sheets on bladder smooth muscle regeneration, the cell sheets were prelabeled with CMFDA. And immunofluorescence staining showed that most of the desmin‐positive SMCs simultaneously expressed CMFDA in the pedicled construct group at 3 months after reconstruction, which meant that the regenerated smooth muscle mainly originated from the SMC sheets (Figure 7d–g).

Furthermore, immunohistochemical staining for S100β revealed regeneration of nerve cells in the smooth muscle layer in the pedicled construct group at 3 months after reconstruction (Figure 7h). Meanwhile, S100β‐positive nerve cells were also apparent in both the capsule tissue layer (Figure 7i) and the submucosal layer (Figure 7j). Thus, the nerve cells in the smooth muscle layer might originate from the buccal submucosa or the capsule tissue.

### 2.7 Morphology and function of the reconstructed bladders

**FIGURE 8:**
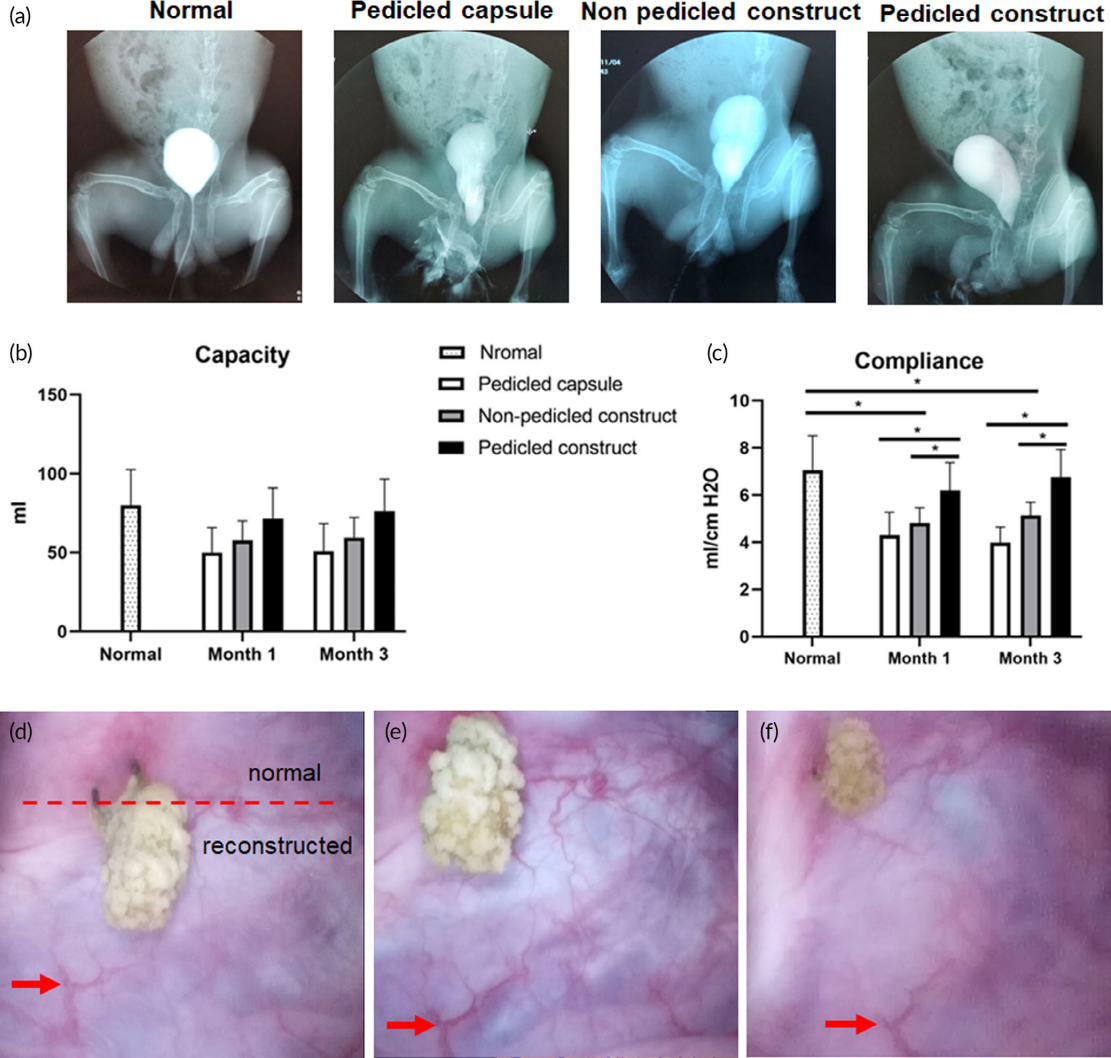
Morphology and function of the normal and reconstructed bladders. (a) Cystography of normal bladder and reconstructed bladders at 3 months after reconstruction. (b) Comparison of bladder capacity. (c) Comparison of bladder compliance. (d–f) Ductility of the reconstructed mucosa observed under cystoscopy at 3 months after reconstruction. The distance between the marked vessel (red arrow) and the calculus gradually increased during the process of water infusion. The data are shown as the mean ± SD; *p < 0.05.

Bladder morphology was evaluated by cystography. The results demonstrated that the morphology was somewhat irregular in the pedicled capsule group but basically normal in the nonpedicled construct group and pedicled construct group compared with normal bladder (Figure 8a). Bladder capacity and compliance were measured by urodynamic studies. The bladder capacities all declined to some extent in the three groups compared with normal bladder (highest decline in the pedicled capsule group and lowest decline in the pedicled construct group) at both 1 and 3 months after reconstruction, but the difference was not statistically significant (Figure 8b). This might be attributed to the fact that the bladder defect was not large enough. Instead, significant declines in bladder compliance were observed in the pedicled capsule group and nonpedicled construct group at both 1 and 3 months but not in the pedicled construct group (Figure 8c). In other words, only the pedicled construct could effectively maintain bladder compliance after bladder reconstruction. Finally, the ductility of the reconstructed bladder mucosa was detected under cystoscopy. In the pedicled construct group, the reconstructed bladder mucosa was slightly paler than the normal site at 3 months after reconstruction (Figure 8d) but exhibited good ductility during the process of water infusion (Figure 8d–f).

## 3 DISCUSSION

In urology, many conditions such as bladder cancer, neurogenic bladder, and bladder exstrophy require reconstruction. Therapeutic options for these severe bladder diseases are limited.^25^ The first clinical bladder substitution by a bowel segment can be traced back to 1913, and it has become a standard method in clinical treatment.^26^ The ileum is most frequently used due to its better compliance and less contractility compared with other segments. However, this kind of method not only requires the sacrifice of autologous intestinal segments but also results in large amounts of complications. The early complications mainly include ileus, anastomotic bowel leakage, infection, urinary leakage, renal failure, hematuria, ureteric obstruction, bleeding, and so on. Late complications involve urolithiasis, uretero‐intestinal stricture, renal function decline, metabolic disorder, urinary incontinence, urinary retention, and sexual dysfunction.^26^ Thus, there is an urgent need for a new strategy for bladder reconstruction with less invasion and complications.

An engineered construct with anatomic and functional characteristics of normal bladder tissue would lead to structural reconstruction and functional restoration. In the previous study, we were able to generate cellularized smooth muscle constructs that contain perfusable vascular networks and pedicle, leading to contractile function.^20^ In this study, we fabricated full‐layer bladder constructs by adding the buccal mucosa onto the pedicled vascularized smooth muscle constructs. The results strongly suggested that the addition of buccal mucosa onto the vascularized smooth muscle constructs resulted in full integration, good survival, and maintenance of barrier function. Furthermore, implantation of the pedicled vascularized full‐layer bladder constructs was able to facilitate the restoration of normal anatomy and function to a large extent in rabbit bladder defects.

For successful reconstruction of full‐thickness bladder defects by bioengineered constructs, a continuous mucosal layer with barrier function is critical. Since hypertonic and cytotoxic urine has been proven to be destructive to the survival of implanted cells, developing a strategy to achieve effective barrier function of the engineered constructs before implantation is of prime importance.^10^ However, this has not been realized in most relevant studies. For scaffold‐based strategies, only scaffolds with low porosity have effective resistance against urine penetration; unfortunately, they may also inhibit seeded cell distribution and migration.^10^ On the other hand, epithelial cell‐based strategies also encounter problems such as difficulty of expanding in vitro and need to be cocultured with 3T3 fibroblasts.^27^ This restricts its clinical usability. In addition, uroepithelial cells from diseased bladders exhibit weaker proliferation and differentiation than normal cells.^28^ In this study, we demonstrated that the buccal mucosa could integrate with the vascularized smooth muscle construct and provide an effective barrier function to protect the inner SMCs. The preformed vasculature improved the survival of the construct and the recovery of bladder function.

The buccal mucosa is a nonkeratinized epithelium and possesses a barrier function to protect against potential pathogens and toxic substances.^29^, ^30^ Its physical barrier function mainly consists of stratified epithelial cells, cell–cell junctions, and certain lipids to maintain permeability.^30^, ^31^ Buccal mucosa is frequently used for urethral reconstruction in clinical practice.^32^, ^33^ A few studies have also demonstrated that buccal mucosa is an appropriate substitution for bladder mucosa with fewer adverse histopathological findings after long‐term exposure to urine.^24^ It has also been shown that the oral keratinocytes within the buccal mucosa possess the potential to transdifferentiate toward urothelial cells in the local bladder microenvironment.^34^ Thus, BMG has been clinically used for reconstruction of the bladder neck.^35^

Therefore, we investigated the feasibility of fabricating a full‐layer bladder construct by combining BMG with bioengineered vascularized smooth muscle tissue on the capsule vascular bed. The results showed that the six‐layer SMC sheets could form vascularized smooth muscle tissue after cultivation on the capsule vascular bed for 1 week, and the BMG with partial submucosa could integrate well with the vascularized smooth muscle construct after cocultivation for another week. Meanwhile, the mucosal layer of the newly formed multilayer construct maintained its barrier function with the continuous expression of ZO‐1, claudin‐1, and occludin, which are key proteins of tight junctions in epithelial cells.^36^ According to these results, we fabricated a full‐layer bladder construct that consisted of mucosal layer, submucosal layer, smooth muscle layer, and capsule tissue layer, structurally imitating native bladder tissue. The construct showed high proliferative activity and adequate vascularization with a vascular pedicle. In addition, the submucosa contained abundant vascular vessels and nerve cells, which might be beneficial for the growth and functional recovery of the construct.

To validate the pedicled vascularized bladder constructs in vivo, they were implanted to repair bladder defects in rabbits. The outcomes revealed good vascularization and effective structural recovery. Structural recovery was confirmed by consecutive mucosal layer and increased smooth muscle content with appropriate collagen content, which was comparable to native bladder tissue. Regeneration of nerve cells in the smooth muscle layer was observed, which probably originated from the submucosa and needs to be proven by further experiments. We previously demonstrated that the pedicled smooth muscle constructs accelerated vascularization after implantation, which was also apparent in this study. The pedicled vascularized bladder constructs resulted in better vascular density than the nonpedicled vascularized constructs, which also led to inconsecutive mucosa, poor smooth muscle content, and severe fibrosis. In terms of functional recovery, the pedicled vascularized bladder constructs exhibited good ductility of mucosa and better bladder compliance compared with the pedicled capsule group and nonpedicled construct group. Although the contractibility of the engineered bladder construct was not examined in this study, it was previously assessed in the vascularized smooth muscle construct.^20^ We speculated that integration of the buccal mucosa would not result in significant changes in the contractibility of the vascularized smooth muscle.

Although encouraging results were achieved in this study, there were still some limitations. The major disadvantage was that the autologous engineered bladder construct was not large enough to imitate the actual clinical situations, and the key limitation was the available size of the buccal mucosa. To overcome this problem, we are attempting to enlarge the area of the BMG by special means, such as mincing it into small particles and making it regrow into an integrated tissue in vivo.^37^ The ultimate purpose is to create larger bladder constructs to realize extensive bladder reconstruction in future studies. In addition, multiple surgeries may increase the risks of infection, hemorrhage anesthesia accidents, and other unexpected circumstances. Thus, we are trying to optimize the whole process and make it as simple as possible. Finally, the follow‐up time was not long enough. Although the short‐term results are encouraging, long‐term follow‐up is still essential to evaluate the long‐term histological and functional changes as well as expose late complications.

## 4 MATERIALS AND METHODS

### 4.1 Animals

Twenty‐eight New Zealand white male rabbits weighing 2.5–3.0 kg (provided by the Department of Laboratory Animal Science, School of Medicine, Shanghai Jiao Tong University) were included in this study. Four rabbits were used as normal control. The remaining 24 rabbits were randomly divided into three groups with eight rabbits each: (1) pedicled capsule group (pedicled capsule tissue only), (2) nonpedicled construct group (vascularized bladder construct without pedicle), and (3) pedicled construct group (pedicled vascularized bladder construct). The rabbits were anesthetized with an intravenous injection of 20–30 mg/kg sodium pentobarbital, and surgeries were performed under sterile conditions. The whole animal experimental protocol was approved by the Animal Care and Use Committee of Shanghai Jiao Tong University School of Medicine. The experimental workflow is presented in Figure 1.

### 4.2 Induction of capsule vascular bed

A sterile spherical skin expander (15 ml) was implanted adhere to the separated SCIs in the inguinal region. After wound closure (approximately 7 days later), the expander was repeatedly inflated with 3 ml of saline solution at 2‐day intervals until the amount was increased to 15 ml. One week after the expander was fully expanded, the tissue expander was obtained for histological analyses. After the sample was embedded in paraffin and cut into 4 μm‐thick sections, hematoxylin and eosin (HE) staining and immunohistochemistry were performed according to standard protocols. For immunohistochemistry, the sections were blocked with donkey serum for 30 min and incubated with CD31 antibody (1:500; Novus, USA).

### 4.3 Preparation of SMC sheets

In vitro culture of SMCs and EPCs and fabrication of SMC sheets were performed as previously described.^18^, ^20^, ^38^ Three cell sheets were stacked together by aspirating one cell sheet with media and attaching it to the basal cell sheet sequentially, as we reported before.

### 4.4 Fabrication of vascularized smooth muscle tissue

The capsule tissue was opened, and the expander was removed temporarily. Triple‐layer cell sheets were first placed on the top of a transparent polypropylene sheet and then transplanted onto the capsule vascular bed by sliding from the polypropylene sheet. After the fully filled expander was placed back into the capsule pouch, the incisions were closed with 4‐0 nylon sutures. Two days later, another triple‐layer cell sheet construct was repeatedly transplanted onto the previous cell sheets and continued to be cultivated for another 7 days. To evaluate the role of EPCs in angiogenesis in vivo, EPCs were labeled with 10 mmol/L CellTracker Green CMFDA before coculturing with SMCs and fabricating cell sheets.

### 4.5 Angiogenesis of vascularized smooth muscle tissue

At 3 and 7 days after transplantation of six‐layer SMC sheets on the capsule vascular bed, a PAM, named Hadatomo™ Z WEL5200 (ADVANTEST, Japan), was used for real‐time three‐dimensional imaging of vascular vessels within the vascularized smooth muscle tissue. The probe was placed on the corresponding superficial skin, and the vascularized smooth muscle tissue was first scanned by grayscale ultrasonography to obtain the boundaries. Then, three‐dimensional images of the microvessels were scanned under the following settings: frequency: 500 MHz, wavelength: 532 nm. Immediately after PAM scanning, vascularized smooth muscle tissue was obtained for immunofluorescence (CD31, 1:500; Novus, USA).

### 4.6 Fabrication of vascularized bladder construct

After six‐layer SMC sheets were cultivated on the capsule vascular bed for 7 days, a suitable BMG with partial submucosal tissue was separated by infiltrating normal saline into the inferior surface of the cheek and placed on the vascularized smooth muscle tissue. The fully filled expander was placed back into the capsule pouch, and the incisions were closed with 4‐0 nylon sutures. The implanted tissues were allowed to cultivate for another 7 days to form a full‐layer vascularized bladder construct with a vascular pedicle.

### 4.7 Histology of the pedicled vascularized bladder construct

After incision of the skin and capsule pouch, the expander was removed, and the bladder construct along with surrounding capsule tissue was exposed. To examine the perfusability of the bladder construct, the ipsilateral femoral artery was separated and injected with 20 ml heparinized Indian ink. Then the construct was harvested and placed in 4% paraformaldehyde. After dehydration and embedding, the tissue was cut into 4 μm‐thick sections for HE staining, immunohistochemistry, and immunofluorescence according to standard staining protocols. Antibodies against AE1/AE3 (1:500; Invitrogen, USA), CD31 (1:500; Novus, USA), desmin (1:100; Invitrogen), alpha‐smooth muscle actin (α‐SMA; 1:500; Abcam, England) and Ki67 (1:500; Abcam) were used for immunohistochemistry. For immunofluorescence, CD31 (1:500; Novus) was applied. In addition, normal bladder tissue was obtained for HE and immunohistochemistry staining.

### 4.8 Bladder reconstruction

The cell sheets were labeled with 10 mmol/L CellTracker Green CMFDA to verify the effects of the SMC sheets on bladder regeneration. Depending on the shape and diameter of the engineered bladder construct, a full‐thickness resection with a mean diameter of 1.8 cm was made on the anterior wall of the empty bladder in each rabbit. The defective bladders were then, respectively, reconstructed by pedicled capsule tissue, nonpedicled vascularized bladder construct, and pedicled vascularized bladder construct. In the pedicled capsule group, the capsule tissue (1 month after full expansion) was isolated on its vascular pedicle trimmed to the corresponding size. The pedicled capsule flap was then pulled from the groin space to the abdominal cavity and sutured onto the bladder defect. In the nonpedicled construct group and pedicled construct group, the vascularized bladder constructs were isolated from the capsule vascular beds without or with vascular pedicle and transferred into abdominal cavities to repair the bladder defects. The pedicled capsule tissue and pedicled construct were pulled into abdominal cavities through an incision on the ipsilateral abdominal wall. All the flaps were sutured to the bladder wall using 6‐0 nylon sutures, and incisions were secured with 4‐0 PDS sutures. Postoperatively, for each rabbit, the catheter was left indwelling for 14 days, and prophylactic cefuroxime sodium was administered intravenously for 5 days (0.5 g/day).

At 1 and 3 months after bladder reconstruction, the morphology, function, and histological characteristics of the reconstructed bladders were evaluated.

### 4.9 Morphological and functional evaluation

The urodynamic study was performed according to previously described methods.^20^ Briefly, after general anesthetization, the rabbit's bladder was manually emptied by abdominal pressure. Then a sterile 8Fr transurethral polyurethane catheter was inserted retrograde into the bladder and connected to a pressure transducer in line with an infusion pump (Duet Logic, Medtronic, Denmark). Normal saline was infused at a uniform speed, and pressure/flow measurements were continuously recorded (Duet Logic, Medtronic, Denmark). Bladder compliance was calculated as ∆V/∆P. Among them, ∆V (ml) was the maximal bladder capacity defined as the volume of infusion that triggered the first urine leakage from the urethral orifice. And ∆P (cm H_2_O) was the difference between the threshold pressure triggering voiding and the resting bladder pressure. After urodynamic study, meglumine diatrizoate (Haipu, China) was injected into the bladder, and the bladder morphology was scanned under x‐ray.

### 4.10 Histological analysis

All the samples were fixed in 4% paraformaldehyde followed by embedding and sectioning. Then PSR staining and immunohistochemical staining were performed according to standard protocols. For immunohistochemical staining, antibodies against AE1/AE3 (1:500; Invitrogen), CD31 (1:500; Novus), desmin (1:100; Invitrogen), alpha‐smooth muscle actin (α‐SMA; 1:500; Abcam), and S100β (1:200; Novus) were applied. In addition, an antibody against desmin (1:100; Invitrogen) was used for immunofluorescence. The results were quantified by Image‐Pro Plus 5.1 software for each tissue sample.

### 4.11 Statistical analysis

All statistical analyses were conducted by GraphPad Prism 6.0 software. The data were presented as the mean ± SD. Analysis of variance and Kruskal–Wallis tests were used to determine the differences among the three groups at each time point. A *p* value < 0.05 was considered statistically significant.

## 5 CONCLUSIONS

In summary, in the present study, we fabricated a full‐layer pedicled vascularized bladder construct by integrating buccal mucosa with a vascularized smooth muscle construct and successfully applied it to bladder reconstruction. The approach described here could overcome the challenge of bioengineering fully vascularized bladder constructs with organized structures and primary functions, and it could be easily scaled up to larger constructs in future preclinical experiments.

## AUTHOR CONTRIBUTIONS

**Mingming Yu:** Data curation (lead); formal analysis (lead); methodology (lead); project administration (equal); writing – original draft (lead). **Jiasheng Chen:** Data curation (equal); methodology (equal). **Lin Wang:** Formal analysis (equal); methodology (equal). **Yichen Huang:** Investigation (equal); methodology (equal). **Hua Xie:** Investigation (equal); resources (equal). **Yu Bian:** Software (lead). **Fang Chen:** Conceptualization (lead); funding acquisition (lead); project administration (lead); resources (lead); writing – review and editing (lead).

## CONFLICT OF INTEREST

All authors declare no potential conflicts of interest.

### PEER REVIEW

The peer review history for this article is available at https://publons.com/publon/10.1002/btm2.10440.

## Data Availability

The main data supporting the findings of this study are available within the paper and its Supplementary information. The associated raw data are available from the corresponding author upon reasonable request.

## References

[1] Serrano‐Aroca Á , Vera‐Donoso CD , Moreno‐Manzano V . Bioengineering approaches for bladder regeneration. Int J Mol Sci. 2018;19(6):1796‐1821. doi:10.3390/ijms19061796 29914213 PMC6032229

[2] Zhang F , Liao L . Long‐term follow‐up of neurogenic bladder patients after bladder augmentation with small intestinal submucosa. World J Urol. 2020;38(9):2279‐2288. doi:10.1007/s00345-019-03008-x 31712957

[3] Joseph DB , Borer JG , De Filippo RE , Hodges SJ , McLorie GA . Autologous cell seeded biodegradable scaffold for augmentation cystoplasty: phase II study in children and adolescents with spina bifida. J Urol. 2014;191(5):1389‐1395. doi:10.1016/j.juro.2013.10.103 24184366

[4] Schaefer M , Kaiser A , Stehr M , Beyer HJ . Bladder augmentation with small intestinal submucosa leads to unsatisfactory long‐term results. J Pediatr Urol. 2013;9(6 Pt A):878‐883. doi:10.1016/j.jpurol.2012.12.001 23332207

[5] Caione P , Boldrini R , Salerno A , Nappo SG . Bladder augmentation using acellular collagen biomatrix: a pilot experience in exstrophic patients. Pediatr Surg Int. 2012;28(4):421‐428. doi:10.1007/s00383-012-3063-0 22350082

[6] Atala A , Bauer SB , Soker S , Yoo JJ , Retik AB . Tissue‐engineered autologous bladders for patients needing cystoplasty. Lancet. 2006;367(9518):1241‐1246. doi:10.1016/s0140-6736(06)68438-9 16631879

[7] Chua ME , Farhat WA , Ming JM , McCammon KA . Review of clinical experience on biomaterials and tissue engineering of urinary bladder. World J Urol. 2020;38(9):2081‐2093. doi:10.1007/s00345-019-02833-4 31222507

[8] Schäfer FM , Stehr M . Tissue engineering in pediatric urology ‐ a critical appraisal. Innov Surg Sci. 2018;3(2):107‐118. doi:10.1515/iss-2018-0011 31579774 PMC6604568

[9] Laschke MW , Vollmar B . Vascularization, regeneration and tissue engineering. Eur Surg Res. 2018;59(3–4):230‐231. doi:10.1159/000492372 30244243

[10] Adamowicz J , Pokrywczynska M , Van Breda SV , Kloskowski T , Drewa T . Concise review: tissue engineering of urinary bladder; we still have a long way to go? Stem Cells Transl Med. 2017;6(11):2033‐2043. doi:10.1002/sctm.17-0101 29024555 PMC6430044

[11] Kant RJ , Coulombe KLK . Integrated approaches to spatiotemporally directing angiogenesis in host and engineered tissues. Acta Biomater. 2018;69:42‐62. doi:10.1016/j.actbio.2018.01.017 29371132 PMC5831518

[12] Zhou F , Zhang L , Chen L , et al. Prevascularized mesenchymal stem cell‐sheets increase survival of random skin flaps in a nude mouse model. Am J Transl Res. 2019;11(3):1403‐1416. 30972170 PMC6456548

[13] Nagase K , Nagumo Y , Kim M , et al. Local release of VEGF using fiber mats enables effective transplantation of layered cardiomyocyte sheets. Macromol Biosci. 2017;17(8). doi:10.1002/mabi.201700073 28547766

[14] Utzinger U , Baggett B , Weiss JA , Hoying JB , Edgar LT . Large‐scale time series microscopy of neovessel growth during angiogenesis. Angiogenesis. 2015;18(3):219‐232. doi:10.1007/s10456-015-9461-x 25795217 PMC4782613

[15] Epple C , Haumer A , Ismail T , et al. Prefabrication of a large pedicled bone graft by engineering the germ for de novo vascularization and osteoinduction. Biomaterials. 2019;192:118‐127. doi:10.1016/j.biomaterials.2018.11.008 30448696

[16] Rnjak‐Kovacina J , Gerrand YW , Wray LS , et al. Vascular pedicle and microchannels: simple methods toward effective In vivo vascularization of 3D scaffolds. Adv Healthc Mater. 2019;8(24):e1901106. doi:10.1002/adhm.201901106 31714024

[17] Guo HL , Wang L , Jia ZM , et al. Tissue expander capsule as an induced vascular bed to prefabricate an axial vascularized buccal mucosa‐lined flap for tubularized posterior urethral reconstruction: preliminary results in an animal model. Asian J Androl. 2020;22(5):459‐464. doi:10.4103/aja.aja_133_19 31929196 PMC7523609

[18] Jia Z , Guo H , Xie H , et al. Construction of Pedicled smooth muscle tissues by combining the capsule tissue and cell sheet engineering. Cell Transplant. 2019;28(3):328‐342. doi:10.1177/0963689718821682 30712374 PMC6425107

[19] Guo HL , Jia ZM , Wang L , et al. Tubularized urethral reconstruction using a prevascularized capsular tissue prelaminated with buccal mucosa graft in a rabbit model. Asian J Androl. 2019;21(4):381‐386. doi:10.4103/aja.aja_43_19 31267985 PMC6628739

[20] Guo HL , Peng XF , Bao XQ , et al. Bladder reconstruction using autologous smooth muscle cell sheets grafted on a pre‐vascularized capsule. Theranostics. 2020;10(23):10378‐10393. doi:10.7150/thno.47006 32929355 PMC7482816

[21] Fry CH , McCloskey KD . Spontaneous activity and the urinary bladder. Adv Exp Med Biol. 2019;1124:121‐147. doi:10.1007/978-981-13-5895-1_5 31183825

[22] Rajasekaran M , Stein P , Parsons CL . Toxic factors in human urine that injure urothelium. Int J Urol. 2006;13(4):409‐414. doi:10.1111/j.1442-2042.2006.01301.x 16734860

[23] Jaimes‐Parra BD , Valle‐Díaz de la Guardia F , Arrabal‐Polo M , et al. Ex vivo construction of a novel model of bioengineered bladder mucosa: a preliminary study. Int J Urol. 2016;23(1):85‐92. doi:10.1111/iju.12963 26502190

[24] Filipas D , Fisch M , Fichtner J , et al. The histology and immunohistochemistry of free buccal mucosa and full‐skin grafts after exposure to urine. BJU Int. 1999;84(1):108‐111. doi:10.1046/j.1464-410x.1999.00079.x 10444136

[25] Stein R , Zahn K , Huck N . Current indications and techniques for the use of bowel segments in pediatric urinary tract reconstruction. Front Pediatr. 2019;7:236. doi:10.3389/fped.2019.00236 31245339 PMC6581750

[26] Thakare N , Lamb BW , Biers S . Orthotopic bladder substitution: surgical aspects and optimization of outcomes. BJUI Compass. 2021;2(6):359‐369. doi:10.1002/bco2.84 35474698 PMC8988640

[27] Utheim TP , Utheim ØA , Khan QE , Sehic A . Culture of oral mucosal epithelial cells for the purpose of treating limbal stem cell deficiency. J Funct Biomater. 2016;7(1):5‐22. doi:10.3390/jfb7010005 26938569 PMC4810064

[28] Subramaniam R , Hinley J , Stahlschmidt J , Southgate J . Tissue engineering potential of urothelial cells from diseased bladders. J Urol. 2011;186(5):2014‐2020. doi:10.1016/j.juro.2011.07.031 21944117

[29] Dawson DV , Drake DR , Hill JR , Brogden KA , Fischer CL , Wertz PW . Organization, barrier function and antimicrobial lipids of the oral mucosa. Int J Cosmet Sci. 2013;35(3):220‐223. doi:10.1111/ics.12038 23320785 PMC3640763

[30] Şenel S . An overview of physical, microbiological and immune barriers of oral mucosa. Int J Mol Sci. 2021;22(15):7281‐7295. doi:10.3390/ijms22157821 34360589 PMC8346143

[31] Wertz PW . Roles of lipids in the permeability barriers of skin and oral mucosa. Int J Mol Sci. 2021;22(10):5229‐5243. doi:10.3390/ijms22105229 34063352 PMC8155912

[32] Soave A , Kluth L , Dahlem R , et al. Outcome of buccal mucosa graft urethroplasty: a detailed analysis of success, morbidity and quality of life in a contemporary patient cohort at a referral center. BMC Urol. 2019;19(1):18. doi:10.1186/s12894-019-0449-5 30885184 PMC6421675

[33] Osman NI , Hillary C , Bullock AJ , MacNeil S , Chapple CR . Tissue engineered buccal mucosa for urethroplasty: progress and future directions. Adv Drug Deliv Rev. 2015;82‐83:69‐76. doi:10.1016/j.addr.2014.10.006 25451857

[34] Lu M , Zhou G , Liu W , et al. Remodeling of buccal mucosa by bladder microenvironment. Urology. 2010;75(6):1514.e7‐1514.e14. doi:10.1016/j.urology.2009.12.060 20394977

[35] Avallone MA , Quach A , Warncke J , Nikolavsky D , Flynn BJ . Robotic‐assisted laparoscopic subtrigonal inlay of buccal mucosal graft for treatment of refractory bladder neck contracture. Urology. 2019;130:209. doi:10.1016/j.urology.2019.02.048 31063762

[36] Samiei M , Ahmadian E , Eftekhari A , Eghbal MA , Rezaie F , Vinken M . Cell junctions and oral health. EXCLI J. 2019;18:317‐330. doi:10.17179/excli2019-1370 31338005 PMC6635732

[37] Reinfeldt Engberg G , Chamorro CI , Nordenskjöld A , Fossum M . Expansion of submucosal bladder wall tissue in vitro and in vivo. Biomed Res Int. 2016;2016:5415012. doi:10.1155/2016/5415012 27777947 PMC5062021

[38] Jia Z , Guo H , Xie H , et al. Harvesting prevascularized smooth muscle cell sheets from common polystyrene culture dishes. PLoS One. 2018;13(9):e0204677. doi:10.1371/journal.pone.0204677 30256839 PMC6157888

